# *BRCA1*/*2* mutations in GI cancers: the risk to Pakistani families

**DOI:** 10.1097/MS9.0000000000000171

**Published:** 2023-03-16

**Authors:** Shahzaib Samad, Aysha Khan

**Affiliations:** Jinnah Sindh Medical University, Karachi, Pakistan

**Keywords:** *BRCA1*, *BRCA2*, GI cancer, mutation, Pakistan

## Abstract

An article on the incidence of *BReast CAncer gene 1*/*2* mutations in Pakistan to those of ovarian, breast, and other cancers, as well as their newly found association to gastrointestinal cancers, and the incidence and mortality rates of these malignancies. It involves a perspective on the epidemiological significance of *BReast CAncer gene 1*/*2* mutations to various cancers and their immense risk to Pakistani families, as well as suggestions to tackle the problem, including possible advancement in their detection and treatment options to improve mortality rates.

Cancer is one of the most prevalent causes of mortality worldwide, resulting in nearly 10 million deaths in 2020, or about one in six deaths, according to WHO^1^. Evidence suggests that early detection and treatments are essential in reducing cancer morbidity and improving the chances of overall patient survival^2^. One method of early diagnosis is genetic testing. *BReast CAncer gene 1* (*BRCA1*) and *BReast CAncer gene 2* (*BRCA2*) are tumor suppressor genes that contribute to DNA repair and transcriptional regulation in response to DNA damage and are required for chromosomal stability. In contrast, their pathogenic variants make an individual more susceptible to cancers. The clinical significance of genetic testing for *BRCA1* and *BRCA2* genes in breast, ovarian, prostate, and pancreatic cancers is already well known and has been evaluated extensively in the last decades. However, their role in other malignancies is still not well established.

Our article highlights that the pathogenic variants of *BRCA* genes could also have a predisposing role in gastrointestinal (GI) cancers since recent studies have specifically proven a new relationship between *BRCA1*/*2* mutations and GI cancer types. A case–control analysis conducted by Momozawa *et al*. identified clinical characteristics, and the link of family history in patients possessing pathogenic *BRCA* genes. They included DNA samples and clinical information from 63 828 patients with 14 common cancer types. Consequently, 315 pathogenic *BRCA* variants were identified, and an odds ratio of greater than 4.0 were found for biliary tract cancer in *BRCA1*, esophageal cancer in *BRCA2*, and gastric cancer in *BRCA1* and *BRCA2* 3.

Another study by Parikh *et al*. examined tumor samples from 17 486 patients with advanced colorectal, gastroesophageal, or small bowel carcinomas. After genomic profiling, genetic alterations were found in 17% of the cases, out of which the third and fourth most commonly mutated genes of the series were *BRCA2* (2.3%) and *BRCA1* (1.1%)^4^. This additionally proved that *BRCA1*/*2* mutations are an important risk factor for GI cancers. Moreover, according to a meta-analysis, *BRCA2* mutation carriers had an increased risk for gastric, esophageal, and biliary tract cancers but not significantly with any other GI cancers. However, the results of this analysis did not find a significant association between *BRCA1* mutations and GI outcomes^5^. These contradictory findings make it difficult to reach a valid conclusion, but they do signify a positive correlation between *BRCA1*/*2* mutations and various GI cancers and indicate the need to perform further research.

Genetic testing is a medical test that analyzes mutations in genes, chromosomes, or proteins. Its results can confirm a suspected genetic condition or help determine a person’s chance of developing or passing on a genetic disorder^6^. Interestingly, many studies showed that patients with pathogenic *BRCA* variants were more likely to report a family history of the associated cancer types; hence, early identification of individuals who carry germline mutations is not only useful for patients with cancer but also for their relatives at risk^7^. Currently, treatment options in advanced GI cancers include radiotherapy, surgical intervention, immunotherapy, and targeted therapy, all of which have proven efficacy in gastric cancers^8^. Moreover, chemotherapeutic agents are also used extensively. However, these drugs are unable to discriminate between normal and cancer cell types, and thus the patients commonly experience adverse side effects such as nausea, vomiting, constipation, and diarrhea^9^.

Contrarily, targeted therapies directly affect cancer-specific mutations to inhibit tumor growth and progression, while minimizing the effects on surrounding nonmalignant tissues, resulting in more favorable patient outcomes. Poly adenosine diphosphate-ribose polymerase (PARP) inhibitors are a good example of targeted anticancer therapy as they function by inhibiting the action of PARP enzymes which help in DNA repair, signal transduction, telomere maintenance, transcriptional regulation, and so on. Their efficacy in *BRCA* gene mutations was first observed in breast and ovarian cancer patients, and later in prostate and pancreatic cancers, implicating a possible further use in the treatment of *BRCA*-linked GI cancers^10^.

Over the last decade, the prevalence of cancer in Pakistan has grown at an alarming rate, with 178 388 new cases detected in 2020^11^, leading to detrimental effects on its already compromised healthcare system and socioeconomic status. Furthermore, Pakistan has the highest incidence of breast and ovarian cancers among Asian countries, with an annual rate of breast cancer of 69.1 per 100 000^12^. This is also outlined by the substantial incidence of *BRCA1*/*BRCA2* mutations among Pakistani families, accounting for 24.7% of high-risk breast cancer patients in the country^13^. While these mutations are currently linked primarily to malignancies of the breast and ovaries, the risk of GI cancers, among which colorectal, gastric, and esophageal cancers are among the most common cancers in Pakistani men^14^.

Multiple nongovernmental organizations from all over Pakistan are providing financial assistance as well as free-of-cost treatment to the affected patients^15^, but this is far from enough as many cancer patients in Pakistan are still lacking access to timely diagnosis and treatment. Moreover, genetic testing and PARP inhibitors are neither easily available nor economical for most of the population. To ease the burden of this national catastrophe, strong measures are needed to be taken by the government, such as funding research to renew policies regarding cancer management, offering financial support to the patients, and making diagnostic and treatment facilities more affordable and accessible to the masses. Furthermore, new epidemiological studies must be conducted to accurately determine the risk factors of the various cancers in Pakistani families, as well as their relations to other cancers and possible genetic anomalies. This new data can thus help shape a more concrete plan to identify families and patients at risk of not only GI, but also breast, ovaries, and pancreatic cancers and help diagnose them at an early stage, where mortality rates are low.

## Ethical approval

This paper did not involve patients; therefore, no ethical approval was required.

## Patient consent

This study was not done on patients or volunteers; therefore, no written consent was required.

## Sources of funding

No funding was secured for this study.

## Author contribution

S.S. and A.K.: conception of the study, drafting of the work, final approval, and agreeing to the accuracy of the work; also reviewed and revised the manuscript.

## Conflicts of interest disclosure

The authors have no conflicts of interest relevant to this article to disclose.

## Research registration unique identifying number (UIN)

1. Name of the registry: not applicable.
2. Unique identifying number or registration ID: not applicable.
3. Hyperlink to your specific registration (must be publicly accessible and will be checked): not applicable.

## Guarantor

Shahzaib Samad and Aysha Khan.

## References

[1] WHO. Cancer. Accessed 11 October 2022. https://www.who.int/news-room/fact-sheets/detail/cancer

[2] Crosby D Bhatia S Brindle KM . Early detection of cancer. Science 2022;375:eaay9040. 35298272 10.1126/science.aay9040

[3] Momozawa Y Sasai R Usui Y . Expansion of cancer risk profile for *BRCA1* and *BRCA2* pathogenic variants. JAMA Oncol 2022;8:871–878. 35420638 10.1001/jamaoncol.2022.0476 PMC9011177

[4] Parikh AR He Y Hong TS . Analysis of DNA damage response gene alterations and tumor mutational burden across 17,486 tubular gastrointestinal carcinomas: implications for therapy. Oncologist 2019;24:1340–1347. 31040255 10.1634/theoncologist.2019-0034 PMC6795150

[5] Lee Y-C Lee Y-L Li C-Y . *BRCA* genes and related cancers: a meta-analysis from epidemiological cohort studies. Medicina (Kaunas) 2021;57:905. 34577828 10.3390/medicina57090905 PMC8464901

[6] MedlinePlus. What is genetic testing?. Accessed 11 October 2022. https://medlineplus.gov/genetics/understanding/testing/genetictesting/

[7] Stoffel EM . Screening in GI cancers: the role of genetics. J Clin Oncol 2015;33:1721–1728. 25918283 10.1200/JCO.2014.60.6764

[8] Joshi SS Badgwell BD . Current treatment and recent progress in gastric cancer. CA Cancer J Clin 2021;71:264–279. 33592120 10.3322/caac.21657 PMC9927927

[9] Nurgali K Jagoe RT Abalo R . Editorial: Adverse effects of cancer chemotherapy: anything new to improve tolerance and reduce sequelae? Front Pharmacol 2018;9:245. 29623040 10.3389/fphar.2018.00245 PMC5874321

[10] Rose M Burgess JT O’Byrne K . PARP inhibitors: clinical relevance, mechanisms of action and tumor resistance. Front Cell Dev Biol 2020;8:564601. 33015058 10.3389/fcell.2020.564601 PMC7509090

[11] WHO. Population fact sheet – burden of cancer in Pakistan. https://gco.iarc.fr/today/data/factsheets/populations/586-pakistan-fact-sheets.pdf.

[12] Rashid MU Zaidi A Torres D . Prevalence of *BRCA1* and *BRCA2* mutations in Pakistani breast and ovarian cancer patients. Int J Cancer 2006;119:2832–2839. 16998791 10.1002/ijc.22269

[13] Ahmad Z Idrees R Fatima S . Commonest cancers in Pakistan – findings and histopathological perspective from a premier surgical pathology center in Pakistan. Asian Pac J Cancer Prev 2016;17:1061–1075. 27039726 10.7314/apjcp.2016.17.3.1061

[14] Rashid MU Muhammad N Naeemi H . Spectrum and prevalence of *BRCA1*/*2* germline mutations in Pakistani breast cancer patients: results from a large comprehensive study. Hered Cancer Clin Pract 2019;17:27. 31528241 10.1186/s13053-019-0125-5 PMC6737632

[15] Ali A Manzoor MF Ahmad N . The burden of cancer, government strategic policies, and challenges in Pakistan: a comprehensive review. Front Nutr 2022;9:940514. 35938114 10.3389/fnut.2022.940514 PMC9355152

